# Synergistic Action of Antimicrobial Lung Proteins against *Klebsiella pneumoniae*

**DOI:** 10.3390/ijms222011146

**Published:** 2021-10-15

**Authors:** Víctor Fraile-Ágreda, Olga Cañadas, Timothy E. Weaver, Cristina Casals

**Affiliations:** 1 Department of Biochemistry and Molecular Biology, Complutense University of Madrid, 28040 Madrid, Spain; vicfra01@ucm.es; 2 Division of Pulmonary Biology, Cincinnati Children′s Hospital Medical Center and University of Cincinnati College of Medicine, Cincinnati, OH 45229, USA; timothyeweaver@icloud.com

**Keywords:** multidrug-resistant bacteria, antimicrobial peptides, pulmonary surfactant, SP-A, SP-B^N^, synergy, peptide–membrane interactions, lipid vesicles, membrane probes, differential scanning calorimetry

## Abstract

As key components of innate immunity, lung antimicrobial proteins play a critical role in warding off invading respiratory pathogens. Lung surfactant protein A (SP-A) exerts synergistic antimicrobial activity with the *N*-terminal segment of the SP-B proprotein (SP-B^N^) against *Klebsiella pneumoniae* K2 in vivo. However, the factors that govern SP-A/SP-B^N^ antimicrobial activity are still unclear. The aim of this study was to identify the mechanisms by which SP-A and SP-B^N^ act synergistically against *K. pneumoniae*, which is resistant to either protein alone. The effect of these proteins on *K. pneumoniae* was studied by membrane permeabilization and depolarization assays and transmission electron microscopy. Their effects on model membranes of the outer and inner bacterial membranes were analyzed by differential scanning calorimetry and membrane leakage assays. Our results indicate that the SP-A/SP-B^N^ complex alters the ultrastructure of *K. pneumoniae* by binding to lipopolysaccharide molecules present in the outer membrane, forming packing defects in the membrane that may favor the translocation of both proteins to the periplasmic space. The SP-A/SP-B^N^ complex depolarized and permeabilized the inner membrane, perhaps through the induction of toroidal pores. We conclude that the synergistic antimicrobial activity of SP-A/SP-B^N^ is based on the capability of this complex, but not either protein alone, to alter the integrity of bacterial membranes.

## 1. Introduction

Lower respiratory tract infections are among the most common infectious diseases affecting humans and constitute a leading cause of death worldwide, as suggested by the World Health Organization [1]. The management and treatment of these infections is challenged by the rapid emergence of multidrug-resistant pathogens such as the respiratory pathogen *Klebsiella pneumoniae* [2,3]. *K. pneumoniae* is an opportunistic Gram-negative respiratory pathogen that can cause community and serious nosocomial infections and is associated with high rates of mortality, particularly in immunocompromised patients or patients with mechanical ventilation [4,5,6,7]. The recent emergence and spread of hypervirulent strains of these bacteria, in conjunction with the increase in resistant strains to antibiotics, have made the treatment of these infections a challenge [8,9].

Among the virulence factors that contribute to the pathogenicity of *K. pneumoniae* are bacterial lipopolysaccharide (LPS) and the exopolysaccharide capsule (CPS). LPS is a major constituent of the outer membranes of Gram-negative bacteria and is crucial for bacterial growth and bacterial viability [10]. LPS determines the pathogenesis of the endotoxin shock associated with Gram-negative bacterial infections [11,12,13,14]. This endotoxin is released from bacteria during cell division, cell death, or antibiotic therapy, causing a potent stimulation in the immune system [11,14]. LPS consists of three different regions: Lipid A, a core oligosaccharide region, and the *O*-polysaccharide specific chain, whose composition varies with bacterial species [15]. Lipid A contains the hydrophobic, membrane-anchoring region of LPS. The core oligosaccharide region consists of a short, rather invariable chain of sugars that connects the lipid A anchor to the *O*-specific chain or *O*-antigen. The *O*-antigen is attached to the core oligosaccharide and extends from the core out into the environment. The *O*-antigen is much longer than the core oligosaccharide and contains the hydrophilic domain of LPS. Wild-type enterobacterial species with *O*-chains are termed “smooth,” and their LPS is called “smooth LPS” (S-LPS). Mutants producing LPS lacking *O*-specific chains are termed “rough” (R), and their LPS is designated as Ra, Rb, Rc, Rd, and Re in order of decreasing core length [15]. On the contrary, CPS secreted around the outer membrane of bacteria, also termed antigen K, acts as an external barrier that protects bacteria and mediates the interaction with their environment. These virulence factors mainly contribute to antibiotic resistance by acting as physical barriers against antimicrobial agents and preventing recognition by macrophages, allowing the bacteria to evade the host′s immune system [16,17].

Due to this barrier formed by LPS and CPS, the development of antibiotics for multidrug-resistant bacteria is complicated. The decreasing effectiveness of conventional antimicrobial drugs has potentiated the development of new non-antibiotic therapies. Research on antimicrobial peptides (AMPs) is attracting increasing attention because of their broad-spectrum antibacterial activity, high cell selectivity, lower levels of pathogen resistance, and relatively short amino acid sequence [9,18,19,20]. They can also exert immunomodulatory and adjuvant functions by acting chemotactic for immune cells and by inducing cytokines and chemokines secretion [9,20]. Thus, AMPs could be employed to develop novel antibiotics for the treatment of multidrug-resistant infections.

Among human lung defense antimicrobial proteins, surfactant protein A (SP-A) and the *N*-terminal segment of the SP-B proprotein (SP-B^N^) have attracted considerable interest as a new therapy by acting synergistically against the respiratory pathogen *K. pneumoniae* K2 [21]. SP-B^N^ is an 8 kDa anionic saposin-like peptide derived from the SP-B proprotein, which is secreted into the air space together with surfactant components [22]. In vitro, this peptide directly kills bacteria at an acidic, not neutral, pH. SP-B^N^ indirectly promotes the phagocytosis of bacteria by macrophage cell lines, and SP-B^N^ overexpression in the distal airway epithelium protects mice against infection with *Staphylococcus aureus* and *Pseudomonas aeruginosa* [22]. SP-A is also secreted into the alveolar fluid, where it recognizes a great variety of immune and non-immune ligands [23,24,25]. SP-A binds to surfactant membranes, as well as to membrane receptors present in macrophages, epithelial cells, and lymphocytes, modifying their response to different stimuli. SP-A also recognizes pathogen-associated molecular patterns on some microorganisms and facilitates microbial clearance by macrophages or recruited neutrophils [23,24,25]. It has been demonstrated that SP-A-deficient mice show decreased microbial clearance from the alveolar space and increased tissue markers of inflammation [26], but data supporting the direct antimicrobial activity of SP-A are sparse. Most respiratory pathogenic bacteria and fungi are resistant to SP-A [23,24,25]. However, we discovered that cooperative interactions between SP-A and SP-B^N^ enhance the microbicidal defense of the lungs. Specifically, we found that: (i) The two proteins directly interact (K_d_ = 0.4 ± 0.08 µM), forming aggregates of 950 ± 50 nm; (ii) this interaction confers new antimicrobial properties, including the ability to bind, kill, and enhance phagocytosis of pathogenic *K. pneumoniae* K2 that is otherwise resistant to either protein alone; (iii) this effect is stronger at an SP-A/SP-B^N^ weight ratio of 10:1; and (iv) administration of both proteins in vivo slows the growth of an established *K. pneumoniae* K2 bacterial infection, supporting the clinical relevance of combined therapy [21]. However, the mechanism of action of this synergistic antimicrobial activity is unknown. We hypothesize that SP-A/SP-B^N^ aggregates may disrupt bacterial membranes and loss of barrier function, leading to cell death.

## 2. Results

**Figure 1:**
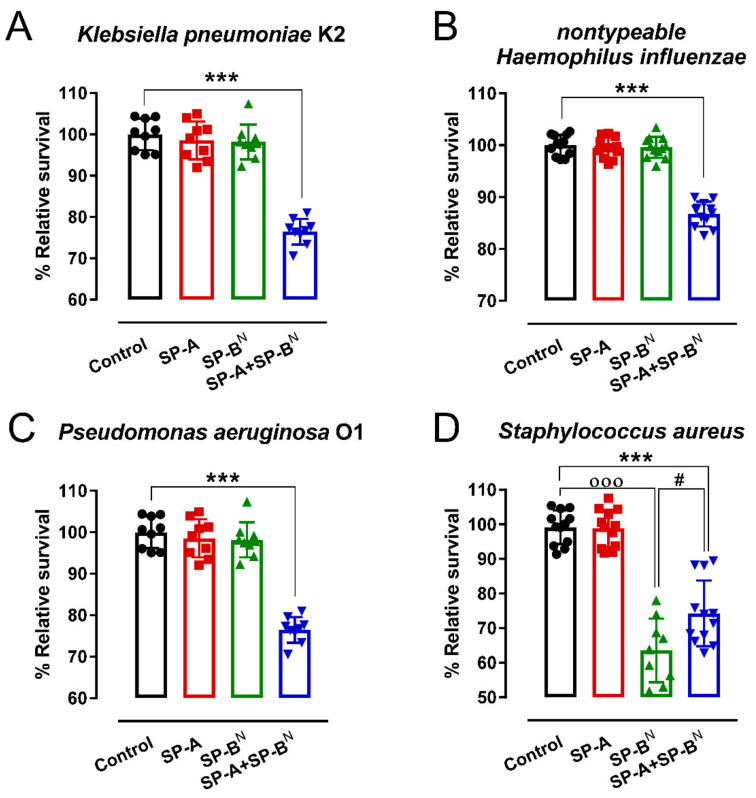
Antimicrobial activity of SP-A and/or SP-B^N^ on *K. pneumoniae* K2 (**A**), non-typeable *Haemophilus influenza* (**B**), *Pseudomonas aeruginosa* O1 (**C**), and *Staphylococcus aureus* (**D**). The bacteria (10^5^ CFU/mL) were incubated for 1 h at 37 °C with SP-A and/or SP-B^N^ in 20 mM phosphate buffer and 150 mM NaCl at pH 7. Then, the bacteria were plated on LB agar for CFU count after 18 h of incubation at 37 °C. The results are shown as % viable bacteria (percentage of live colony counts compared with untreated control) and are the mean ± SD of at least nine experiments. A value of *p* < 0.001 was obtained for the one-way ANOVA, followed by the general multiple-comparison Bonferroni test: In (**A**–**C**), *** *p* < 0.001 when comparing SP-A+SP-B^N^ treatment vs. the control and either the SP-A- or SP-B^N^-treated group. In (**D)**, *** *p* < 0.001 when comparing SP-A+SP-B^N^ treatment vs. the control and SP-A-treated groups; *# p* < 0.5 when comparing SP-A+SP-B^N^ treatment vs. the SP-B^N^-treated group; ooo *p* < 0.001 when comparing SP-B^N^ treatment vs. the control untreated group.

Figure 1 shows that neither SP-B^N^ nor SP-A alone was able to kill capsulated *K. pneumoniae* K2 at neutral pH, as previously reported [22], or other Gram-negative bacteria that express the whole structure of LPS such as *Pseudomonas aeruginosa* O1 or non-typeable *Haemophilus influenza*; however, in combination, SP-A and SP-B^N^ effectively killed Gram-negative bacteria (Figure 1A–C). SP-B^N^ showed direct antimicrobial activity against the Gram-positive bacterium *Staphylococcus aureus*, and the SP-A/SP-B^N^ complex was not more effective than SP-B^N^ alone against *S. aureus* (Figure 1D). In the next experiments, we investigated the effect of SP-A and SP-B^N^ on the integrity of *K. pneumoniae* K2 membranes and the effect of these proteins on model membranes of the inner and outer Gram-negative bacterial membranes.

### 2.1. Effect of SP-A and SP-B^N^ on the Integrity of K. pneumoniae Membranes

**Figure 2:**
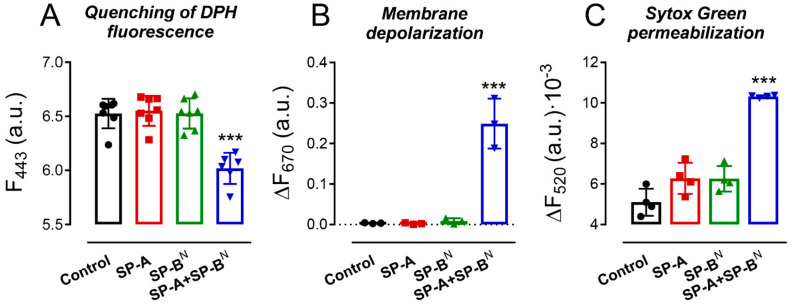
The SP-A/SP-B^N^ complex, but not the proteins alone, altered the integrity of the outer and cytoplasmic membranes of *K. pneumoniae*. Bacteria were incubated with SP-A and/or SP-B^N^ in the presence of 1,6-diphenyl-1,3,5-hexatriene (DPH) (**A**), 3,3′-dipropylthiadicarbocyanine iodide (DiSC_3_-(5)) (**B**), or Sytox Green (**C**), and the change in the fluorescence of the dyes was recorded as a function of time. Data shown in (**A**) correspond to the fluorescence of DPH after treatment with the proteins for 30 min, whereas (**B**,**C**) show the difference in the fluorescence of the dyes at t = 30 min and t = 0 min. The experiments were performed at 37 °C. Protein concentration: SP-A 154 nM (100 µg/mL) and SP-B^N^ 1.25 µM (10 µg/mL). The results are the mean ± SD of at least three experiments, each one triplicated. A *p*-value < 0.001 was obtained for the overall one-way ANOVA (Bonferroni-corrected *p*-value: *** *p* < 0.001 when compared to the control).

We evaluated the ability of SP-A and/or SP-B^N^ to alter the integrity of *K. pneumoniae* K2 membranes by using the fluorescent dye 1,6-diphenyl-1,3,5-hexatriene (DPH), a fluorescent probe that incorporates into the hydrophobic core of lipid bilayers. DPH has a low quantum yield and a very short lifetime when exposed to water. Thus, its fluorescence is sensitive to the amount of water that penetrates the lipid bilayer [27]. Figure 2A shows that SP-A or SP-B^N^ alone did not affect the fluorescence of the dye, while the SP-A/SP-B^N^ mixture significantly decreased the dye’s fluorescence, indicating the presence of water molecules in the microenvironment of the bacterial membranes of *K. pneumoniae* K2 (Figure 2A).

We then investigated the ability of the SP-A/SP-B^N^ complex to depolarize the bacterial cytoplasmic membrane. The depolarization of the membrane is a step prior to membrane permeabilization and bacterial death. To determine the potential loss of a membrane, we used 3,3′-dipropylthiadicarbocyanine iodide, DiSC_3_-(5), a membrane potential-dependent probe that increases its fluorescence when a bacterial membrane is depolarized. As shown in Figure 2B, SP-A or SP-B^N^ alone did not affect the fluorescence of the dye, whereas the addition of the SP-A/SP-B^N^ mixture significantly increased the dye’s fluorescence, indicative of depolarization of the cytoplasmic membrane of *K. pneumoniae* K2. Since this study was performed without removal of the outer membrane, the observed changes in the fluorescence of DiSC_3_-(5) may reflect the interaction of the probe with both the inner and the outer membranes. The ability of the SP-A/SP-B^N^ mixture to permeabilize *K. pneumoniae* K2 membranes was further assessed by using the fluorescent dye Sytox Green, a dye whose fluorescence is enhanced upon binding to DNA once the cytoplasmic membrane is compromised [28]. The addition of SP-A or SP-B^N^ alone did not statistically affect the fluorescence of the dye, while the SP-A/SP-B^N^ mixture significantly increased the dye’s fluorescence, indicative of the permeabilization of the cytoplasmic membrane of *K. pneumoniae* K2 (Figure 2C). These results agree with the absence of binding of SP-A and SP-B^N^ individually to *K. pneumoniae* and the incapability of either protein alone to kill the bacteria [21].

**Figure 3:**
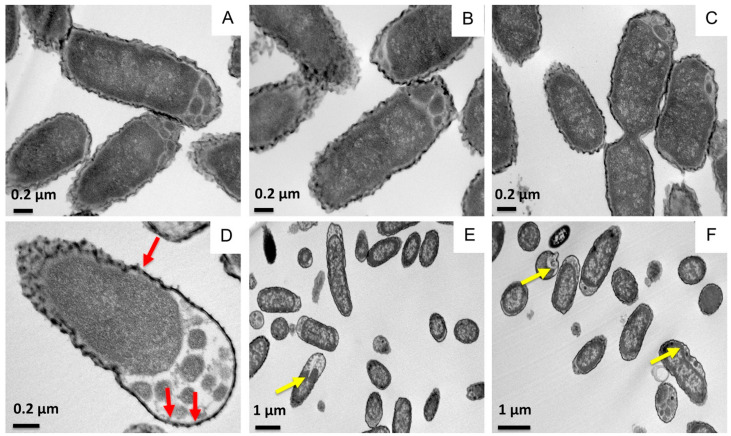
Effect of SP-A and/or SP-B^N^ on the ultrastructure and morphology of *K. pneumoniae* K2. Untreated bacteria (**A**), and bacteria treated with SP-A (100 µg/mL) (**B**), SP-B^N^ (10 µg/mL) (**C**), or SP-A+SP-B^N^ (**D**–**F**). Red arrows indicate pores in the bacterial outer membrane, while yellow arrows indicate protrusions in the cytoplasmic membrane. One representative experiment of three is shown.

To further characterize the effect of the SP-A/SP-B^N^ complex on *K. pneumoniae* membranes, we recorded transmission electron microscopy (TEM) images of the bacteria in the absence and presence of the proteins. Untreated (Figure 3A) and treated bacteria with either SP-A (100 µg/mL) (Figure 3B) or SP-B^N^ (10 µg/mL) (Figure 3C) showed conserved morphology with intact outer and cytoplasmic membranes. However, bacteria treatment with the SP-A/SP-B^N^ complex (10:1 weight ratio) (Figure 3D–F) produced significant ultrastructural changes: (i) Localized swelling in polar regions; (ii) altered bacterial membrane integrity and accumulation of osmium tetroxide in the vicinity of stain-depleted regions, which could have been induced by the formation of potential pores in the outer membrane; and (iii) formation of numerous small vesicles surrounded by the cytoplasmic membrane. In addition, treatment with the SP-A/SP-B^N^ complex produced the appearance of a bacterial population with a larger size than untreated bacteria or treated with SP-A and SP-B^N^ alone (Table 1), indicating that the SP-A/SP-B^N^ complex produced a significant increase in bacterial length.

### 2.2. Effect of SP-A and SP-B^N^ on Bacterial Model Membranes

To gain insight into the mechanism of action of the SP-A/SP-B^N^ complex, we next evaluated the effect of both proteins, alone or in conjunction, on model membranes that mimicked the outer and inner membranes of Gram-negative bacteria. The inner membrane consisted of a mixture of Gram-negative bacterial phospholipids (BPL): Phosphatidylethanolamine/phosphatidylglycerol/cardiolipin (70:20:10, *w/w/w*). The outer membrane was composed of bacterial lipopolysaccharide, Re-LPS, and bacterial phospholipids at a Re-LPS/BPL molar ratio of 1.2:1. We used the serotype Re-LPS, instead of S-LPS, since the presence of the *O*-antigen in the S-LPS molecule increases the water solubility of LPS, preventing the incorporation of the lipopolysaccharide into mixed vesicles of LPS with BPL.

**Figure 4:**
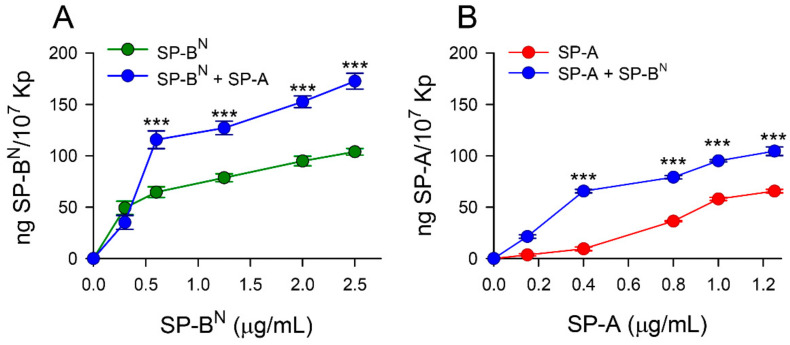
Binding capacity of SP-A and/or SP-B^N^ to non-capsulated deep rough *K. pneumoniae* strains (Re-LPS Kp-CPS). (**A**) The concentration of biotinylated SP-B^N^ associated with Re-LPS Kp-CPS was measured by a solid phase binding assay in the presence and absence of SP-A (10 μg/mL). (**B**) Binding capacity of biotinylated SP-A to Re-LPS Kp-CPS in the presence and absence of SP-B^N^ (10 μg/mL). The results are the mean ± SD of three independent experiments. A value of *** p < 0.001 was obtained for the one-way ANOVA, followed by the general multiple-comparison Bonferroni test.

To substantiate the use of Re-LPS in the bacterial membrane models, we evaluated the binding capacity of both biotinylated SP-A and SP-B^N^ to non-capsulated deep rough *K. pneumoniae* strains (Figure 4). Our results indicate that SP-B^N^ bound to the Re-LPS Kp-CPS strains in a concentration-dependent manner (from 0 to 2.5 µg/mL) and the addition of SP-A (10 µg/mL) significantly increased the binding of biotinylated SP-B^N^ to mutant bacteria that express Re-LPS (Figure 4A). Likewise, the binding of biotinylated SP-A (from 0 to 1.25 µg/mL) was determined in the presence and absence of SP-B^N^ (10 µg/mL) to these mutant bacteria that expresses Re-LPS. Again, biotinylated SP-A bound to Re-LPS Kp-CPS strains in a concentration-dependent manner and the binding of biotinylated SP-A significantly increased in the presence of SP-B^N^ (Figure 4B).

**Figure 5:**
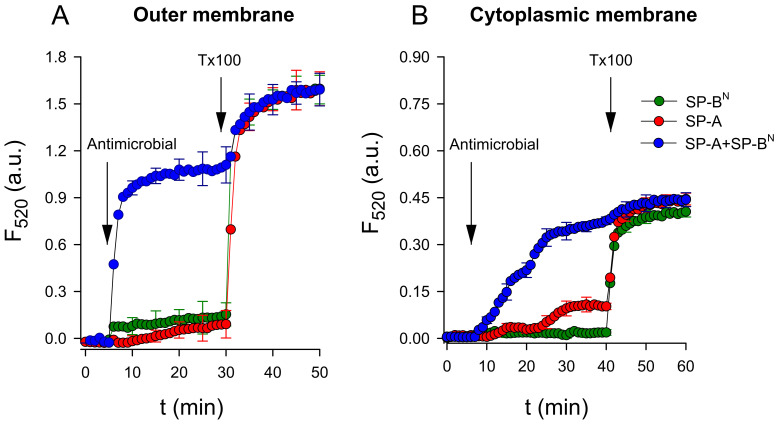
SP-A/SP-B^N^-induced a leakage of 8-aminonapthalene-1,3,6 trisulfonic acid (ANTS)/p-xylene-bis-pyridinium bromide (DPX) from large unilamellar vesicles (LUVs) that mimic the outer (**A**) and cytoplasmic (**B**) membranes of *K. pneumoniae*. In all experiments, SP-A (154 nM) and/or SP-B^N^ (1.25 µM) was added to a cuvette containing 75 µM of lipid vesicles that entrapped the fluorescent probe ANTS and its quencher DPX. The leakage of ANTS and DPX increased the fluorescence due to decreased quenching. Triton X-100 was added at 30 min (for outer membrane model vesicles) or 40 min (for cytoplasmic membrane model vesicles) to completely disrupt the vesicles and release 100% of the contents. The experiments were performed at 25 °C with λ_ex_ = 353 nm and λ_em_ = 520 nm. The results are the mean ± SD of three experiments. A *p*-value < 0.001 was obtained for the overall one-way ANOVA (Bonferroni-corrected *p*-value: *p* < 0.001 when compared to LUVs treated with SP-A or SP-B^N^ individually).

Next, we evaluated the ability of the SP-A/SP-B^N^ complex and the individual proteins (SP-A or SP-B^N^) to permeabilize the inner and outer model bacterial membranes consisting of BPL and Re-LPS/BPL, respectively. To this end, we used large unilamellar vesicles that mimicked both the outer and inner bacterial membranes, which entrapped the fluorescent probe 8-aminonapthalene-1,3,6 trisulfonic acid (ANTS) and its quencher p-xylene-bis-pyridinium bromide (DPX). Figure 5 shows that the addition of SP-A or SP-B^N^ to both model membranes had no effect on the fluorescence of the dye. However, the addition of the SP-A/SP-B^N^ complex increased ANTS fluorescence in both model membranes (Figure 5A,B). A comparison of these results with those obtained for the permeabilization of *K. pneumoniae* using the fluorescent probe Sytox Green indicates that these membrane models reproduced the effects observed in bacteria.

#### 2.2.1. The Outer Membrane

**Figure 6:**
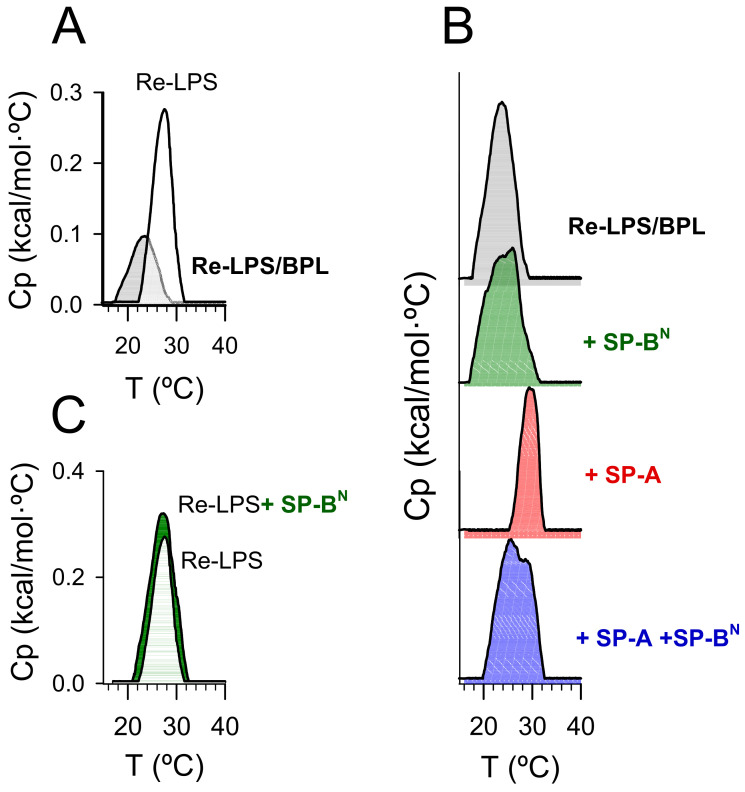
DSC trace of multilamellar vesicles composed of Re-LPS/BPL or pure Re-LPS aggregates (**A**). The effect of SP-B^N^ 1.25 µM (10 µg/mL), SP-A 154 nM (100 µg/mL), or SP-A/SP-B^N^ on the DSC trace of multilamellar vesicles composed of Re-LPS/BPL (**B**). The effect of SP-B^N^ 1.25 µM (10 µg/mL) on the DSC trace of pure Re-LPS aggregates (**C**). Calorimetric scans were performed at a rate of 0.5 °C/min. In (**B**), the tick labels of heat capacity (Cp) are not shown for clarity of presentation. For each thermogram, the scale of Cp was from 0 to 0.12. One representative experiment of three experiments is shown.

To find out the mechanism by which SP-A/SP-B^N^ permeabilizes the outer membrane of Gram-negative bacteria, we studied the effect of SP-A and SP-B^N^, alone or in conjunction, on the thermotropic behavior of multilamellar vesicles composed of Re-LPS/BPL (1.2:1 by mol) by differential scanning calorimetry (DSC) (Figure 6 and Table 2).

Figure 6A compares the thermotropic behavior of Re-LPS/BPL multilamellar vesicles (0.6 mM) to that of pure Re-LPS aggregates (0.4 mM). The Re-LPS/BPL vesicles showed a single-phase transition, suggestive of lipid miscibility. Compared to the Re-LPS aggregates, the phase transition of Re-LPS/BPL vesicles exhibited a broader peak, with a T_m_ value (23.5 ± 0.2 °C) between those corresponding to pure BPL (< 0 °C) and pure Re-LPS (28.69 ± 0.01 °C) and a smaller enthalpy (0.6 ± 0.1 kcal/mol for Re-LPS/BPL vesicles vs. 1.2 ± 0.1 kcal/mol for pure Re-LPS).

The addition of SP-B^N^ to Re-LPS/BPL vesicles broadened the transition and induced the appearance of a shoulder on the high-temperature side of the transition (Figure 6B), indicative of peptide-induced lipid phase segregation. The fact that the shoulder appeared on the high-temperature side of the transition suggests an interaction of the peptide with Re-LPS, the lipid with a higher phase transition temperature [29]. To evaluate whether this effect may be due to the binding of SP-B^N^ to Re-LPS, we analyzed the effect of the peptide on the DSC trace of Re-LPS aggregates (Figure 6C). We found that SP-B^N^ substantially increased the transition enthalpy of Re-LPS (2.0 ± 0.1 kcal/mol vs. 1.2 ± 0.1 kcal/mol for pure Re-LPS) but did not affect the Tm (28.68 ± 0.01 °C). This effect is characteristic of hydrophilic proteins that interact with lipids through electrostatic forces [29]. Therefore, our results suggest that SP-B^N^ binds to Re-LPS, inducing some demixing of Re-LPS and BPL.

On the contrary, the addition of SP-A concentrations up to 154 nM (100 µg/mL) shifted the transition to higher temperatures and decreased the transition enthalpy (Figure 6B and Table 2). We previously found that SP-A strongly binds to LPS, recognizing its lipid A portion and promoting Re-LPS extraction of the membrane [30,31,32]. The reduction in the transition enthalpy of Re-LPS/BPL membranes is in agreement with SP-A-mediated Re-LPS extraction. In addition, the results of Figure 6B suggests that SP-A promotes the segregation of Re-LPS, decreasing Re-LPS miscibility with bacterial phospholipids.

The interaction of the SP-A/SP-B^N^ complex with Re-LPS/BPL vesicles was different to that of each protein alone. The SP-A/SP-B^N^ complex shifted the thermogram upward, broadened the DSC trace, and induced the appearance of a shoulder on the high-temperature side of the transition (Figure 6B). This effect is characteristic of proteins that interact with more ordered lipids [29]. This suggests the preferential interaction of the SP-A/SP-B^N^ complex with the bacterial lipopolysaccharide, which may induce segregation of membrane lipids into different domains and lipid packing defects that would render the membrane leaky.

**Figure 7:**
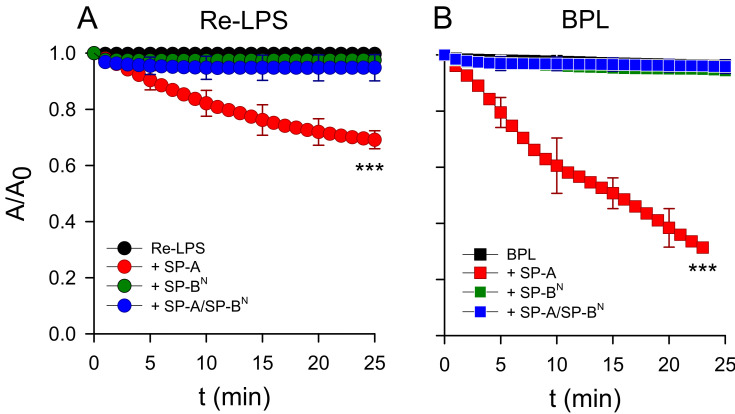
Effect of SP-A (1.54 nM) and/or SP-B^N^ (12.5 nM) on the relaxation kinetics of the Re-LPS (**A**) and BPL (**B**) monolayers spread onto a saline subphase during t = 30 min at a constant surface pressure (32 mN/m). The temperature of the subphase was 25.0 ± 0.1 °C. The results are the mean ± SD of three experiments. A *p*-value < 0.001 was obtained for the overall one-way ANOVA (Bonferroni-corrected *p*-value: *** *p* < 0.001 when compared to the control).

To demonstrate that the mode of interactions of the SP-A/SP-B^N^ complex are different to that of SP-A alone, which can extract Re-LPS from monolayers as previously described [30], we performed relaxation kinetics studies at a constant surface pressure of the monolayers of Re-LPS (Figure 7A) and BPL (Figure 7B). Figure 7 shows that both lipid monolayers were stable over time, while the addition of a small amount of SP-A (1.54 nM) in the subphase caused a decrease in the number of lipid molecules in both monolayers, confirming SP-A′s activity of extracting lipid molecules from monolayers, forming three-dimensional structures [30]. However, the addition of SP-B^N^ (12.5 nM) and the SP-A/SP-B^N^ complex did not produce changes in the relaxation kinetics, as the lipid monolayers were stable over time in both films (Figure 7A,B). These results suggest that the permeabilization of bacterial membranes of *K. pneumoniae* K2 by the SP-A/SP-B^N^ complex is only based on the ability of this complex to produce lipid phase segregation and packing defects into the bacterial membranes deduced by differential scanning calorimetry. The ability of SP-A to extract lipid molecules from membranes is abrogated when it forms a complex with SP-B^N^.

**Figure 8:**
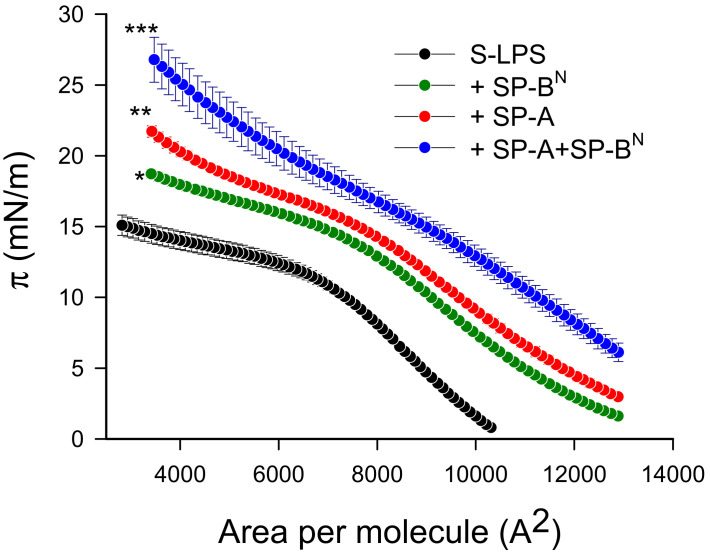
Compression isotherm for the effect of SP-A (1.54 nM) and/or SP-B^N^ (12.5 nM) on the surface pressure (π) vs. area isotherms of pure S-LPS monolayers. The temperature of the subphase was 25.0 ± 0.1 °C. A barrier speed of 50 cm^2^/min was chosen as the optimum velocity for the monolayer compression. The results are the mean ± SEM of three experiments. A *p*-value < 0.001 was obtained for overall one-way ANOVA (Bonferroni-corrected *p*-value: * *p* < 0.05, ** *p* < 0.01, and *** *p* < 0.001 when compared to the control).

Re-LPS lacks *O*-antigen and the outer region of the core. This facilitates the interaction of SP-A and maybe SP-B^N^ with the lipid A portion of LPS. Thus, we evaluated the potential interaction of these proteins, alone or in conjunction, with S-LPS by studying their effect on the compression isotherms of pure S-LPS monolayers. Figure 8 shows that S-LPS formed unstable monolayers, which collapsed at very low surface pressures (15 mN/m), as previously described [32]. Considering that these proteins do not absorb to a clean air/liquid interface, the protein-induced displacement of the S-LPS isotherm to higher molecular areas (Figure 8) indicates that both SP-A and SP-B^N^ incorporated in the monolayer, where SP-A can interact with the lipid A moiety of LPS [32]. The larger displacement of the compression isotherm and the increase in surface pressure observed after injection of the SP-A/SP-B^N^ mixture (Figure 8) are suggestive of a higher incorporation and deeper penetration of the SP-A/SP-B^N^ complex in the S-LPS monolayer.

#### 2.2.2. The Inner Membrane

To study the mechanism by which SP-A/SP-B^N^ alters the permeability of the inner bacterial membrane, we explored the effect of both proteins on the liquid crystalline (L_α_) to inverted hexagonal (H_II_) phase transition (L_α_-H_II_) of palmitoyloleoylphosphatidylethanolamine (POPE) multilamellar vesicles. POPE is a component of the bacterial cytoplasmic membrane and tends to form non-lamellar structures with negative curvature due to the small size of its polar head and the presence of unsaturated acyl chains. 

**Figure 9:**
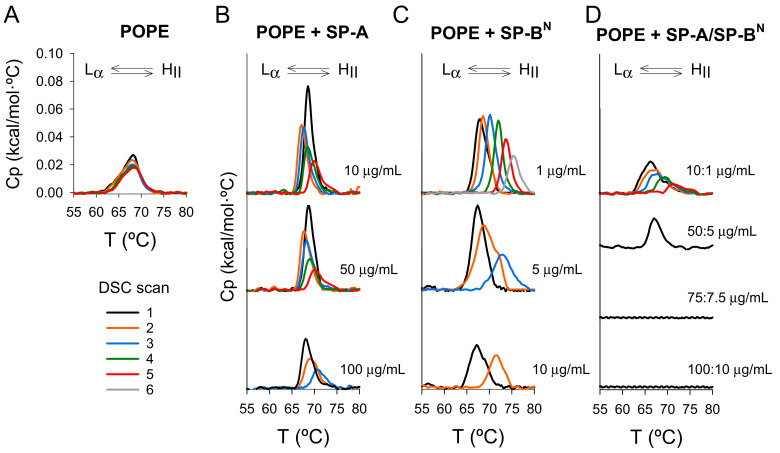
Effect of heating/cooling cycles on liquid crystalline/inverted hexagonal (L_α_–H_II_) phase transition of multilamellar vesicles of palmitoyloleoylphosphatidylethanolamine (POPE) (**A**) and after treatment with SP-A (**B**), SP-B^N^ (**C**), and SP-A/SP-B^N^ (**D**) in a dose-dependent manner. When heating/cooling cycles and the concentration of SP-A and/or SP-B^N^ increased, there was greater incorporation of both molecules into multilamellar vesicles of POPE, causing the inhibition of the L_α_–H_II_ transition and induction of positive curvature. Calorimetric scans were performed at a rate of 0.5 °C/min. One representative experiment of three experiments is shown.

Our results indicate that the thermotropic properties of POPE did not vary significantly during the heating/cooling cycles in DSC measurements (Figure 9A), while the effect of SP-A and/or SP-B^N^ on the L_α_–H_II_ transition depended on the heating/cooling cycles (Figure 9B–D). Thus, an increase in the phase transition temperature (T_h_) and a decrease in the enthalpy (ΔH) of the transition were observed upon cycling (Figure 9B–D and Table 3), indicating inhibition of the L_α_–H_II_ transition and the induction of positive curvature in the cytoplasmic membrane [33,34,35]. These results suggest that: (i) When SP-A or SP-B^N^ were added to POPE membranes, only a few molecules were incorporated into the membrane before the first cycle, and (ii) the structural changes induced in the membrane by the phase transition during the different heating cycles favored the incorporation of more SP-A or SP-B^N^ molecules, enhancing their effect during the next cycle. In this regard, it is possible that both proteins recognize the negative curvature of the membrane in the inverted hexagonal phase. This hypothesis is sustained by the finding that the effects of the SP-A/SP-B^N^ complex on *K. pneumoniae* were mainly observed in the polar regions, where lipids that promote negative curvature are located [36]. Alternatively, packing defects that occur during the phase transition could favor the incorporation of SP-A and/or SP-B^N^. In fact, SP-A tends to localize in packing defects at the borders between the ordered and disordered phases of interfacial lipid films and bilayers [37,38].

We also observed that this effect was dose-dependent. Thus, increasing the amount of protein added to the POPE membrane inhibited the phase transition in earlier cycles (Figure 9B–D and Table 3). SP-B^N^ exerted a stronger effect than SP-A, promoting a larger increase in the phase transition temperature (*T_h_*) and decreasing the enthalpy of the transition (ΔH) to lower values (Figure 9C and Table 3), suggesting a greater relevance for SP-B^N^ in the perturbation of the cytoplasmic membrane. Finally, the effect of the SP-A/SP-B^N^ complex was more accentuated than that of either protein alone, promoting the disappearance of the transition in earlier cycles. At high concentrations of the SP-A/SP-B^N^ complex, the L_α_–H_II_ transition was inhibited during the first cycle (Figure 9D and Table 3). This indicates that the formation of the SP-A/SP-B^N^ complex facilitates the incorporation of the proteins into the membrane, favoring their interaction with lipids.

Our results suggest a correlation between the ability of SP-A and SP-B^N^ to modulate the curvature of the inner membrane and its permeabilization in *K. pneumoniae* bacteria. In this regard, induction of membrane-positive curvature is required for the formation of toroidal pores [33,35,39] and may explain the SP-A/SP-B^N^-induced fission of the cytoplasmic membrane into small vesicles observed by TEM.

## 3. Discussion

The great increase in hypervirulent and multidrug-resistant strains, the absence of new antibiotics, and the diminished efficacy of potent broad-spectrum antimicrobials have driven the development of new alternative therapies based on antimicrobial proteins and peptides from host defense mechanisms [9,20,40]. Recently, our group determined that SP-A acts synergistically with the anionic peptide SP-B^N^ against the clinical strain of *K. pneumoniae* K2 [21], and herein, we demonstrated that the mechanism of this synergistic activity is based on the disruption of bacterial membranes.

According to the structural characteristics of SP-B^N^, this peptide has a B-type saposin module that facilitates transient or permanent interaction with membranes [41,42] and, like saposin B, it is negatively charged. Thus, the interaction of SP-B^N^ with bacterial membranes and capsules, which have a negative charge, would be pH-dependent. A low pH would trigger the protonation and neutralization of negatively charged residues of SP-B^N^, which would otherwise avoid binding to anionic bacterial capsules or bacterial membranes. On the contrary, SP-A is a large extracellular protein. The primary structure of each subunit of SP-A consists of an *N*-terminal segment containing cysteine residues involved in oligomerization, followed by a collagen-like region, an alpha helical coiled neck region, and a globular CRD with a calcium ion at the lectin site [24,25]. SP-A is intracellularly assembled in multiples of three subunits because of its collagen domain. Its supratrimeric assembly has a umbelliform-shaped structure of six trimers, similar to mannose binding protein or C1q [24,25,43].

SP-A or SP-B^N^ individually were unable to bind and kill *K. pneumoniae* K2, which expresses S-LPS and polysaccharide capsule [21], or to other Gram-negative bacteria that express the whole structure of LPS such as *Pseudomonas aeruginosa* O1 or non-typeable *Haemophilus influenza*. However, each protein bound individually to Re-LPS and S-LPS molecules in bacterial membranes and to non-capsulated *K. pneumoniae* strains that express Re-LPS. The SP-A/SP-B^N^ complex, formed at a neutral but not acidic pH [21], presents different properties to each protein separately. The binding of SP-A to SP-B^N^, which depends on NaCl and pH [21], might trigger the neutralization of negatively charged residues of SP-B^N^. In addition, SP-A/SP-B^N^ complexes form a fiber-like structure that could destabilize the bacterial capsule and/or the outer bacterial membrane. Importantly, SP-B^N^ individually is capable of forming aggregates at an acidic pH, and this oligomeric structure can kill *K. pneumoniae* K2 at an acidic pH [21]. Other antimicrobial peptides (such as protegrine-1, dermaseptin S9, and temporins B and L) can also form fibrillar aggregates [44,45], suggesting a possible connection between the aggregation of antimicrobial peptides and their bactericidal activity.

Our results indicate that the SP-A/SP-B^N^ complex presents the ability to alter the bacterial morphology and ultrastructure of *K. pneumoniae* K2, inducing the formation of potential pores in the outer membrane, as shown by TEM. Permeabilization of the outer bacterial membrane would allow the entrance of water molecules and the translocation of both proteins into the periplasmic space, where they would act on the cytoplasmic membrane. We found that the SP-A/SP-B^N^ complex depolarized and permeabilized the inner membrane of *K. pneumoniae* K2. Because of protein–lipid interactions, the cytoplasmic membrane is broken up into smaller membrane fragments that result in small vesicles encapsulating cytoplasmic portions. During this process, transient pores that permeabilize the cytoplasmic membrane might form, causing its depolarization. These mechanisms can happen rapidly, and the downstream effect leads to bacterial death by loss of membrane depolarization and permeability.

As observed in the experiments with model membranes, our results indicate that the SP-A/SP-B^N^ complex, but not the individual proteins, permeabilizes the outer and cytoplasmic bacterial membranes after binding to LPS and bacterial phospholipids. The DSC experiments suggested that, once in contact with the bacterial outer membrane, the SP-A/SP-B^N^ complex could alter the high molecular packing of the LPS molecules present in the bacterial membrane, causing packing defects that increase their permeability and facilitate the entrance of molecules into the periplasmic space. Consequently, there would be an expansion of periplasmic space, causing the separation of outer and cytoplasmic membranes observed by TEM, which is consistent with previously reported studies for gramicidin S [46]. The main activity of the SP-A/SP-B^N^ complex in the outer and cytoplasmic membranes occurs in polar regions, which are enriched in cardiolipin in Enterobacteria [36,47]. The previous results of our group indicate that SP-A binds with great affinity to cardiolipin, suggesting that the SP-A/SP-B^N^ complex could also act on bacterial membranes through interaction with cardiolipin, as previously observed with the antimicrobial peptides daptomycin and c-WFW [48,49].

Our results are supported by some studies with AMPs that indicate that a new AMP mechanism against bacterial membranes is peptide-induced lipid segregation [50,51,52]. This process is suggested for AMPs that exert their antimicrobial activity only on bacterial membranes [51,53]. This mechanism produces packing defects on bacterial membranes that lead to increased membrane permeability, transient pore formation, and cytoplasmic leakage. Additionally, the perturbation of bacterial membranes by lipid segregation would alter the membrane curvature and membrane fluidity [53], causing a general dysregulation in crucial processes such as bacterial division and bacterial growth. Moreover, the existence of specific microdomains in bacterial membranes with important regulatory functions for the cell would be altered by this mechanism, turning them non-functional for bacteria [36,53].

Once the outer membrane is permeabilized and the SP-A/SP-B^N^ complex is translocated into the periplasmic space, SP-A/SP-B^N^ interacts with the cytoplasmic membrane, inducing positive curvature that facilitates its fissure and permeabilization, as inferred by the (L_α_-H_II_) phase transition of multilamellar vesicles of POPE. The induction of positive curvature may be due to the insertion of amphipathic or hydrophobic domains of both proteins into the membrane. This process alters the membrane lateral pressure profile, increasing the pressure in the interfacial region and decreasing it in the hydrophobic core [54]. To reduce this stress, the membrane can form protrusions with positive curvature [54,55]. In general, interfacial active peptides could promote changes in membrane curvature [55]. For anionic and amphipathic AMPs such as SP-B^N^, hydrophobicity drives the binding into bacterial membranes, and this process leads to an excess of negative charge in the membrane. The electrostatic repulsion of peptide molecules and lipid headgroups induces positive curvature, resulting in membrane vesicularization, as previously observed with the peptide surfactin [56] and other antimicrobial peptides [55,57]. Alternatively, positive curvature may be related to the formation of a rigid protein complex covering the bacterial membrane, resulting in a protrusion that narrows in the lower region as the protein complex assembly increases [33,55]. Thus, the induction of positive curvature in the cytoplasmic membrane by the SP-A/SP-B^N^ complex could lead to membrane fission into small vesicles and the formation of toroidal pores that would promote its depolarization and permeabilization [35,55].

Although bacterial membranes are the main target of AMPs, some evidence indicates that many of them can inhibit some crucial intracellular processes such as protein synthesis, DNA or RNA synthesis, enzyme activity, and cell wall synthesis [19,58]. AMPs can show this intracellular activity with or without permeabilizing bacterial membranes [19]. Cell elongation and local swelling in the polar regions of *K. pneumoniae* K2 induced by the SP-A/SP-B^N^ complex suggest that these proteins could act intracellularly by inhibiting protein synthesis [59], in addition to disrupting bacterial membranes. SP-A has been reported to inhibit protein and RNA synthesis in *Escherichia* *coli* and *K. pneumoniae* bacteria that express rough LPS [60]. Dermcidin, an anionic antimicrobial peptide, inhibits RNA and protein synthesis after its interaction with components of bacterial membranes, but does not disrupt membrane permeabilization [20]. Moreover, proline-rich AMPs show direct killing of bacteria by inhibiting protein synthesis without altering membrane integrity [19,61,62]. Proline-rich AMPs also induce bacterial filamentation by blocking DNA replication and inhibiting the synthesis of proteins involved in septum formation [63]. Since the collagen-like domain of SP-A is rich in proline, it is possible that this protein could inhibit bacterial protein synthesis intracellularly.

In conclusion, in this study, we determined the synergistic mechanism of antimicrobial lung proteins against capsulated *Klebsiella pneumoniae* (serotype K2). Knowledge about the mechanism of endogenous AMPs and the improvement of their interaction properties will contribute to increasing their potential as new antimicrobial therapies against multi-resistant pathogens and to reduce the current crisis in the absence of effective antimicrobial therapies.

## 4. Materials and Methods

### 4.1. Materials

The SP-B^N^ peptide was provided by Professor Timothy Weaver (Cincinnati Children′s Hospital Medical Centre, Cincinnati, OH, USA). The fluorescent dyes DPH, Sytox Green, DiSC_3_-(5), ANTS, and DPX were from Molecular Probes (Eugene, OR USA). Rough lipopolysaccharide (Re 595, Re-LPS) and smooth lipopolysaccharide (S-LPS) from *Salmonella enterica* serotype Minnesota, phosphatidylethanolamine, and cardiolipin were from Sigma-Aldrich. Palmitoyloleoylphosphatidylglycerol and POPE were from Avanti Polar Lipids (Birmingham, AL, USA). The organic solvents used to dissolve the lipids were of HPLC grade.

### 4.2. Bacteria

*Klebsiella pneumoniae* 52145 (serotype K2:O1) was provided by Professor José Antonio Bengoechea (Queen′s University Belfast, UK). Bacteria were grown in Luria–Bertani (LB) broth at 37 °C with continuous shaking to the exponential phase. Bacteria were then harvested, resuspended in PBS, and adjusted to the desired final concentration, as described in [21].

### 4.3. SP-A Isolation

SP-A was isolated from the bronchoalveolar lavage of alveolar proteinosis patients using sequential extraction with n-butanol and octilglucoside [43]. The purity of SP-A was determined by one-dimensional SDS-PAGE in 12% acrylamide under reducing conditions and mass spectrometry. The degree of SP-A oligomerization was assessed by electrophoresis under non-denaturing conditions [43] and analytical ultracentrifugation [64]. SP-A was formed by supratrimeric oligomers of at least 18 subunits. Each subunit had an apparent molecular mass of 36,000 Da.

### 4.4. Bacterial Killing Assay

To evaluate the microbicidal activity of SP-A and SP-B^N^ against respiratory pathogens (*Klebsiella pneumoniae* K2, *Pseudomonas. aeruginosa* O1, non-typable *Haemophilus influenzae,* and *Staphylococcus aureus*), we used colony counts on plate assays. Five microliters of bacterial suspensions in the exponential phase were incubated with SP-A (25 µg/mL for non-typable *H. influenza* or 100 µg/mL for *K. pneumoniae* K2, *P. aeruginosa* O1, and *S. aureus*), SP-B^N^ (10 µg/mL), or combinations of both in 20 mM phosphate and 150 mM NaCl buffer at pH 7.0 for 1 h at 37 °C. In all cases, the final bacterial concentration was 10^5^ CFU/mL. At the end of incubation, bacterial suspensions were plated on LB agar plates and incubated for 18 h at 37 °C. Viable bacteria were enumerated by colony count. The results are expressed as % viable bacteria (percentage of live colony counts compared to the untreated control).

### 4.5. Binding Assay of SP-A and SP-B^N^ to Bacteria

Binding assays were performed with biotinylated SP-A and SP-B^N^, which were prepared as previously described [65]. The structure and functional activity of biotinylated proteins were similar to those of unlabeled SP-A and SP-B^N^. The binding assay of biotinylated proteins to mutant *K. pneumoniae* that express Re-LPS was executed as previously described [21]. In brief, exponential-phase bacteria (10^7^ CFU/mL in 5 mM Tris buffer, pH 7.4, 150 mM NaCl, and 175 μM CaCl_2_) were incubated with several concentrations of biotinylated SP-A (0–1.25 µg/mL) or biotinylated SP-B^N^ (0–2.5 µg/mL) in the presence or absence of SP-B^N^ and/or SP-A by gentle orbital rotation for 30 min at room temperature. In all cases, bacteria were pelleted, washed twice, and resuspended in 200 mL of carbonate buffer 0.1 M at pH 9.5. Controls were performed in the absence of bacteria to estimate nonspecific binding. Protein binding was analyzed by solid-phase binding, as follows: Samples were applied to a 96-well plate MaxiSorp (Nunc, Rochester, NY, USA) and allowed to bind for 1 h at 37 °C. The plate was blocked with 5 mM Tris containing 10% FBS for 1 h at 37 °C. After extensive washing, streptavidin-HRP was added to the wells and incubations were performed for 1 h at room temperature. Biotinylated protein detection was performed by adding the 3,3′,5,5′-tetramethylbenzidine liquid substrate. The colorimetric reaction was halted with 4 M sulfuric acid, and the absorbance was read at 450 nm on an ELISA reader (DigiScan; Asys HiTech GmbH, Eugendorf, Austria). The results obtained were expressed as nanograms of bound protein/10^7^ bacteria.

### 4.6. Bacterial Membrane Permeabilization Assays

The ability of SP-A and SP-B^N^, alone and mixed, to permeabilize the outer and cytoplasmic membranes of *K. pneumoniae* K2 was evaluated by quantifying (i) the fluorescence intensity of DPH and (ii) the uptake of Sytox Green. Exponential-phase bacteria (1 × 10^7^ CFU/mL) were treated with SP-A (100 µg/mL) and/or SP-B^N^ (10 µg/mL) at 37 °C for 30 min in PBS buffer. The suspension was incubated with 20 μM of DPH dissolved in *N*,*N*-dimethylformamide for 1 h at 37 °C in darkness. DPH fluorescence intensity was measured using an SLM-Aminco AB-2 spectrofluorimeter equipped with a thermostated cuvette holder (Thermo Spectronic, Waltham, MA, USA). Quartz cuvettes of 5 mm path length were used. The excitation and emission wavelengths were 350 and 450 nm, respectively [30]. Non-labeled bacteria were used as the background. All experiments were conducted in triplicate.

For the measurement of Sytox Green influx, the probe (1.25 μM) was added to 1 mL of *K. pneumoniae* suspension (2 × 10^7^ CFU/mL) in PBS and the sample was incubated for 15 min in darkness at room temperature. Then, 100 µL of the Sytox Green/bacterial suspension mixture was added to each well of a 96-well black microplate containing SP-A, SP-B^N^, and mixtures thereof (final concentrations of SP-A and SP-B^N^: 100 and 10 µg/mL, respectively, with 1 × 10^7^ CFU/mL of bacteria) and fluorescence was monitored for 30 min at 37 °C in a FLUOstar Galaxy microplate reader (BMG Lab Technologies, Ortenberg, Germany) with excitation and emission wavelengths of 485 and 520 nm, respectively [28]. PBS was used as a negative control, whereas ethanol (70%) was used as a positive control [28]. Non-labeled bacteria were used as the background. All experiments were conducted in triplicate.

### 4.7. Cytoplasmic Membrane Depolarization 

To characterize the effect of SP-A and SP-B^N^ on *K. pneumoniae* membrane potential, we used the fluorescent dye DiSC_3_-(5) [66]. This cationic permeant dye is driven into the cell by negative membrane potential. Once in the cytoplasm, the dye forms non-fluorescent aggregates. Upon cell hyperpolarization, DiSC_3_-(5) fluorescence decreases because of the electrophoretical influx of the dye, which results in further formation of non-fluorescent aggregates. Conversely, when the cell depolarizes, the more positive membrane potential induces an electrophoretic efflux of the dye that increases the fluorescence as the DiSC_3_-(5) complexes disaggregate. Depolarization experiments were performed as described elsewhere [66]. Briefly, 5 μL of DiSC_3_-(5) (2 μM) were added to 350 µL of a bacterial suspension (1 × 10^7^ CFU/mL) and the changes in fluorescence due to membrane potential collapse were monitorized continuously in a SLMAminco AB-2 spectrofluorimeter at 37 °C with excitation at 622 nm and emission at 670 nm. Once the maximal DiSC_3_-(5) incorporation was obtained, as indicated by the decrease in fluorescence due to its accumulation in the cytoplasm, SP-A (100 µg/mL), SP-B^N^ (10 µg/mL), or a mixture of SP-A/SP-B^N^ was added and the changes in fluorescence were recorded for 30 min. PBS was used as a negative control, and valinomycin (20 μg/mL) was used as a positive control of the total collapse of membrane potential.

### 4.8. Transmision Electron Microscopy

The effect of SP-A and SP-B^N^ on the ultrastructure of *K. pneumoniae* 52145 was visualized by means of transmission electron microscopy. TEM preparations were made as described in [67]. *K. pneumoniae* bacteria in the mid-logarithmic growth phase (1 x 10^7^ CFU/mL) were treated with SP-A, SP-B^N^, and mixed SP-A/SP-B^N^ (final SP-A and SP-B^N^ concentration: 100 µg/mL and 10 µg/mL, respectively) at 37 °C for 30 min in PBS buffer. Cells were spun down and PBS medium was removed. Cell pellets were then chemically fixed with 4% paraformaldehyde and 2.5% glutaraldehyde for 4 h at 4 °C and washed three times with PBS. Next, bacteria were post-fixed with 1% osmium tetroxide for 1 h. Samples were then washed thrice with bi-distilled water and dehydrated using sequential exposure to acetone concentrations ranging from 30% to 100% for 15 min at room temperature. Next, infiltration and embedding were performed using Spurr’s resin. The samples were sectioned using an ultramicrotome with a diamond knife and were mounted on copper grids. Samples were examined on a Jeol 1010 electron microscope (JEOL, Tokyo, Japan).

### 4.9. Liposome Preparation

Multilamellar vesicles (MLVs) were prepared by dry film formation and hydration, as described in [65,68]. Briefly, MLVs were formed by evaporating the required amounts of the lipids dissolved in chloroform/methanol 3/1 (*v/v*) to dryness under a stream of nitrogen. Solvent traces were subsequently removed by evacuation under reduced pressure. MLVs were obtained by hydrating the dry lipid films in buffer A (5 mM Tris-HCl, pH 7.4, 150 mM NaCl, and 150 µM of CaCl_2_) and allowing them to swell for 1 h at a temperature above the gel-to-liquid phase transition temperature of lipid mixtures. Large unilamellar vesicles (LUVs) were prepared by extrusion of MLVs through a 100 nm pore-sized polycarbonate membrane. The mean diameter of the resulting LUVs was 130 ± 3 nm, as determined by dynamic light scattering using a Malvern Zetasizer Nano S (Malvern Instruments Ltd., Malvern, UK). For differential scanning calorimetry, Re-LPS was suspended directly in buffer A and allowed to swell for 1 h at 45 °C, as previously described [30]. The final lipid and Re-LPS concentrations of both multilamellar and unilamellar vesicles were assessed by phosphorus determination and quantification of 2-keto-3-deoxyoctulosonic acid, respectively.

### 4.10. Membrane Leakage 

To evaluate the effect of SP-A and SP-B^N^, alone and mixed, on model bacterial membrane permeability, the fluorescent dye ANTS (12.5 mM) and its quencher DPX (45 mM) were encapsulated in LUVs composed of Re-LPS/BPL (8:2 by weight) and bacterial phospholipids [69]. To this end, the dye and quencher were added to the lipid hydration buffer. To increase the encapsulation efficiency, the obtained MLVs were subjected to five cycles of freeze/thaw before extrusion. Non-encapsulated ANTS and DPX molecules were removed by size exclusion chromatography using a Sephadex G100 column. After quantification of lipid concentration, LUVs (75 µM of phospholipid) containing ANTS and DPX were placed into 5 mm quartz cuvettes (350 µL) and the fluorescence increase due to leakage and the subsequent dilution of the quenched dye was monitored as a function of time in the absence and presence of SP-A, SP-B^N^, and a mixture of SP-A/SP-B^N^ (final concentrations of SP-A and SP-B^N^: 100 and 10 µg/mL, respectively) in an SLM-Aminco AB-2 spectrofluorimeter with excitation at 353 nm and emission at 520 nm. Triton X-100 was used as a positive control. The leakage percentage was determined according to Equation (1):

(1) $$\%L=\frac{\left(F_{p}-\;F_{0}\right)}{\left(F_{100}-\;F_{0}\right)}\;\times \;100$$

where *F*_0_ and *F_p_* are the fluorescence of the dye before and after the addition of proteins, respectively, and *F*_100_ is the fluorescence corresponding to 100% leakage, as established by the addition of 0.4% Triton X-100.

### 4.11. Differential Scanning Calorimetry (DSC)

For the DSC studies, MLVs composed of an 8:2 (*w/w*) mixture of Re-LPS/BPL were used to mimic the outer membrane of *K. pneumoniae*, while pure POPE MLVs were used to mimic the cytoplasmic membrane. Calorimetric measurements were conducted in a Microcal VP differential scanning calorimeter (Microcal Inc., Northampton, MA, USA) with a heating rate of 0.5 °C/min, as previously described [30,68]. Lipid samples, in the absence and presence of increasing amounts of SP-A, SP-B^N^, and a mixture of SP-A/SP-B^N^, were loaded into the sample cell (lipid concentration: 1 mg/mL), while 0.8 mL of buffer A were loaded into the reference cell. Measurements were performed between 15 and 60 °C for Re-LPS/BPL and between 50 and 90 °C for POPE. Microcal Origin software was used for data acquisition and analysis. Excess heat capacity functions were obtained after subtraction of the buffer-buffer baseline.

### 4.12. Relaxation Kinetics of the Re-LPS and BPL Monolayers

To determine the effects of SP-A and SP-B^N^ on the relaxation kinetics of Re-LPS and BPL monolayers, surface pressure–area isotherms of Re-LPS and BPL monolayers were measured using a thermostated Langmuir–Blodgett trough (102M micro Film Balance, NIMA Technologies, Coventry, UK) equipped with an injection port and magnetically stirred as described in [30]. The trough, of a total area of 100 cm^2^, was equipped with two symmetrical movable barriers controlled by an electronic device, which allowed barrier movement at constant speed. Re-LPS and BPL dissolved in chloroform/methanol 3:1 (*v/v*) was spread onto buffer A (Tris 5 mM pH 7.4, 150 mM NaCl, and 150 µM of CaCl_2_) subphase to form the monolayers. After 15 min of evaporating the organic solvent, the monolayer was compressed to a preset surface pressure that was kept constant by automatically adjusting the surface area of the trough through the movement of barriers (32 mN/m). Once the desired surface pressure was reached, SP-A (1 µg/mL) and/or SP-B^N^ (0.1 µg/mL) were injected into the subphase and a relaxation curve was obtained by recording the trough surface area for 30 min. All measurements were performed at 25.0 ± 0.1 °C. 

### 4.13. Pressure–Area Isotherms of S-LPS Monolayers

To determine whether SP-A and/or SP-B^N^ interact with S-LPS, the effect of both proteins, alone and mixed, on S-LPS compression isotherms was characterized as described in [32]. Briefly, monolayers of S-LPS were formed in the thermostated 102M micro Film Balance by spreading 10 µL of a concentrated solution of the lipid dissolved in a petroleum ether/chloroform/phenol 7:3:1 (*v/v*) solution over a subphase of buffer A. The organic solvent was allowed to evaporate for 15 min. To evaluate the effect of SP-A (1 µg/mL) and/or SP-B^N^ (0.1 µg/mL), the proteins were injected into the subphase once the monolayer was formed. The monolayer was then compressed at 50 cm^2^/min while changes in surface pressure were monitored. All measurements were performed at 25.0 ± 0.1 °C.

### 4.14. Statistical Analysis

Data are presented as means ± SDs. Differences in the means between groups were evaluated by one-way ANOVA, followed by the Bonferroni multiple-comparison test. For comparison of two groups, Student’s *t*-test was used. An α-level ≤5% (*p* ≤ 0.05) was considered significant.

## 5. Conclusions

In summary, we have demonstrated that the mechanism by which the lung proteins SP-A and SP-B^N^ act synergistically against *K. pneumoniae* is based on the direct interaction of the SP-A/SP-B^N^ complex with the lipids of the outer and inner bacterial membranes. The SP-A/SP-B^N^ complex binds to lipopolysaccharide molecules present in the outer membrane forming packing defects in the membrane that alter the ultrastructure of *K. pneumoniae* and may favor the translocation of both proteins to the periplasmic space. Once in the periplasmic space, the interaction of the SP-A/SP-B^N^ complex with the lipids of the cytoplasmic membrane induces a positive curvature in the membrane that could lead to membrane fission into small vesicles and the formation of toroidal pores that would promote the membrane’s depolarization and permeabilization.

## Figures and Tables

**Table 1:** Bacterial length of untreated *K. pneumoniae* K2 and treated with SP-A 154 nM (100 µg/mL), SP-B^N^ 1.25 µM (10 µg/mL), or the SP-A/SP-B^N^ complex, where a bacterial population with a similar length to the untreated bacteria and another bacterial population with a longer length were observed. The results are the mean ± SD of three independent experiments. A *p*-value < 0.001 was obtained for the overall one-way ANOVA (Bonferroni-corrected *p*-value: *** *p* < 0.001 when compared to untreated bacteria).

Sample	Bacterial Length (µM)
Untreated	1.4 ± 0.2
+SP-A 100 µg/mL	1.3 ± 0.2
+SP-B^N^ 10 µg/mL	1.4 ± 0.2
+SP-A/SP-B^N^ (population 1)	1.4 ± 0.2
+SP-A/SP-B^N^ (population 2)	2.1 ± 0.3 ***

**Table 2:** The effect of SP-A 154 nM (100 µg/mL) and SP-B^N^ 1.25 µM (10 µg/mL), alone or mixed at a SP-A/SP-B^N^ molar ratio of 1:8, on the apparent phase transition temperature ($T_{m}^{\mathrm{app}}$), enthalpy (ΔH), and relative cooperativity (T_1/2_) of multilamellar vesicles composed of Re-LPS/BPL (molar ratio of 1.2:1). Data shown are the mean ± SD of three independent experiments. A *p*-value < 0.001 was obtained for the overall one-way ANOVA (Bonferroni-corrected *p*-value: *** *p* < 0.001 when compared to control multilamellar vesicles of Re-LPS/BPL)

Sample	∆H (kcal/mol)	$T_{m}^{\mathrm{app}}$(°C)	T_1/2_ (°C)
Re-LPS/BPL	0.6 ± 0.1	23.5 ± 0.2	5.9 ± 0.6
Re-LPS/BPL+ SP-A	0.35 ± 0.03 ***	30.1 ± 0.4 ***	4.2 ± 0.2 ***
Re-LPS/BPL + SP-B^N^	0.68 ± 0.02	25.6 ± 0.5 ***	8.6 ± 0.1 ***
Re-LPS/BPL + SP-A/SP-B^N^	0.70 ± 0.08	25.4 ± 0.3 ***	8.6 ± 0.4 ***

**Table 3:** Effect of SP-A, SP-B^N^, and SP-A/SP-B^N^ concentrations and heating/cooling cycles on the liquid–crystalline/inverted hexagonal phase transition temperature ($T_{h}^{\mathrm{app}}$), enthalpy (∆H), and relative cooperativity (T_1/2_) of multilamellar vesicles of POPE. The difference between the initial scan (1) and the final scan prior to the inhibition of the transition is shown (cycle number). Data shown are the mean ± SD of three independent experiments.

Sample	Cycle Number	∆H (kcal/mol)	$T_{h}^{\mathrm{app}}$ (°C)	T_1/2_ (°C)
POPE		0.14 ± 0.01	67.9 ± 0.8	3.7 ± 0.6
+ SP-A 10 µg/mL	1	0.13 ± 0.02	68.5 ± 0.5	1.5 ± 0.4
+ SP-A 10 µg/mL	5	0.07 ± 0.03	69.8 ± 0.9	2.5 ± 0.3
+ SP-A 50 µg/mL	1	0.15 ± 0.02	68.6 ± 0.4	2.0 ± 0.3
+ SP-A 50 µg/mL	5	0.05 ± 0.03	69.8 ± 0.7	2.5 ± 0.4
+ SP-A 100 µg/mL	1	0.11 ± 0.02	68.5 ± 0.4	2.2 ± 0.4
+ SP-A 100 µg/mL	3	0.04 ± 0.02	71.0 ± 1.0	3.2 ± 0.9
+ SP-B^N^ 1 µg/mL	1	0.18 ± 0.01	68.3 ± 0.4	2.5 ± 0.9
+ SP-B^N^ 1 µg/mL	6	0.07 ± 0.02	76.0 ± 1.0	2.5 ± 0.5
+ SP-B^N^ 5 µg/mL	1	0.21 ± 0.01	67.5 ± 0.1	4.1 ± 1.2
+ SP-B^N^ 5 µg/mL	3	0.10 ± 0.01	74.0 ± 2.0	3.6 ± 0.5
+ SP-B^N^ 10 µg/mL	1	0.12 ± 0.02	67.5 ± 0.4	4.0 ± 0.4
+ SP-B^N^ 10 µg/mL	2	0.09 ± 0.01	72.0 ± 1.0	3.5 ± 0.9
+ SP-A/SP-B^N^ 10:1 µg/mL	1	0.11 ± 0.02	66.3 ± 0.4	4.4 ± 0.3
+ SP-A/SP-B^N^ 10:1 µg/mL	4	0.06 ± 0.01	70.0 ± 1.0	3.6 ± 0.7
+ SP-A/SP-B^N^ 50:5 µg/mL	1	0.07 ± 0.02	67.5 ± 0.2	3.0 ± 0.5
+ SP-A/SP-B^N^ 75:7.5 µg/mL	1	No transition		
+ SP-A/SP-B^N^ 100:10 µg/mL	1	No transition		
